# Reported Impacts of Congenital Heart Disease on Functional Outcomes in Adults with Down Syndrome

**DOI:** 10.1007/s00246-025-03979-2

**Published:** 2025-07-31

**Authors:** Stephanie S. Gaydos, Andreana Benitez, Paul J. Nietert, Kimberly E. McHugh, Andrew Atz

**Affiliations:** 1 Division of Pediatric Cardiology, Department of Pediatrics, MUSC Shawn Jenkins Children’s Hospital, Medical University of South Carolina, 10 McClennan Banks Dr. (MSC915), Charleston, SC 29425 USA; 2 Department of Neurology, Medical University of South Carolina, Charleston, SC 29425 USA; 3 Department of Public Health Services, Medical University of South Carolina, Charleston, SC 29425 USA

**Keywords:** Congenital heart disease, Down syndrome, Outcomes

## Abstract

To evaluate whether having congenital heart disease (CHD) mediates community engagement, neuropsychiatric conditions, and quality of life (QOL) in adults with Down syndrome (DS). Cross-sectional survey of adults ages 18–45 years with DS and their caregiver, identified from NIH DS Registry, regional DS groups, and Cardiology clinics. The study survey consisted of adapted instruments (validated or prior use in this cohort) assessing medical history, employment/volunteer experience, QOL, mental health, and caregiver burden. Primary outcome was employment. Outcomes were compared between DS groups with and without CHD via Chi-squared test, *t* tests, and two proportion *Z* tests as appropriate. 287 subjects completed the study. Adults with DS and CHD (n = 104, 36%) reported similar employment rates as those without CHD: 61% and 60%, respectively (*p* = 0.87). Current volunteerism was significantly higher in DS + CHD subjects (32%) compared to DS alone (19%); *p* = 0.01. There were higher rates of stroke and seizure in DS + CHD (p < 0.01 for both), although no significant difference in psychological comorbidities (*p* = 0.67). 69% of all DS adults reported mental health problems. Proxy-reported mean QOL scores were similar between DS groups (p = 0.52). Mild-moderate caregiver burden was reported in both groups, with significantly greater burden reported in guardians of DS + CHD (*p* = 0.03). CHD is not associated with reduced employment or community engagement in adults with DS, despite significantly greater neurologic comorbidities. Similar QOL and mental health issues were reported in adults with DS regardless of having CHD. Findings may convey optimism to families of children born with both health issues.

**Supplementary Information:**

The online version contains supplementary material available at 10.1007/s00246-025-03979-2.

## Introduction

Down syndrome (DS) is the most common genetic cause of intellectual disability with prevalence in the U.S.A. of roughly 12 per 10,000 live births [1]. Individuals with DS have well-characterized patterns of neurodevelopment and exhibit degrees of impairment in cognition, language, behavior, and executive function which often hinder their academic achievement [2–5]. In addition, they have a myriad of concurrent systemic conditions throughout the lifespan, such as hypothyroidism, early-onset dementia, psychiatric comorbidities, obstructive sleep apnea and other pulmonary disease, elevated body mass index, and others, and are at risk for reduced employment and productive community participation in adulthood [6, 7]. These are important considerations with the increased average life expectancy for individuals with DS now to an average of 60 years [8].

One common comorbidity in DS is congenital heart disease (CHD), affecting 40–60% of individuals with DS and often requiring surgical correction in infancy [9]. The most common forms of CHD are considered mild to moderate complexity lesions, specifically atrioventricular septal defects, ventricular septal defects, and atrial septal defects [10]. Cardiac operation (or re-operation) is sometimes necessary in adults with DS and CHD, and the postoperative course can be comparatively more complicated by comorbidities frequently seen in adults with DS, such as pulmonary disease [11]. In general, individuals with CHD alone possess risks of neurodevelopmental impairment, reduced quality of life (QOL), impaired academic achievement and cognitive ability, as well as reduced job participation, particularly those with more complex CHD [12–18]. Concurrent DS has been associated with greater academic impairments among adolescents and young adults with CHD compared to those with CHD alone [19]. Additionally, individuals with DS and those with CHD separately possess greater risks of seizures, stroke, and psychologic disorders than the general population, all of which may jeopardize the capacity for productive societal functioning, educational attainment, employment opportunities, QOL, and may contribute to cumulative morbidity [20–24]. These condition-related factors likely translate into higher health care and societal costs among individuals with both DS and CHD as well as greater caregiver burden.

There is an evidence gap regarding the impact of CHD on employment and other functional outcomes among adults with existing risk factors, such as genetic syndromes. Similarly, little has been studied to assess the impact of certain medical conditions, specifically CHD, on employment in adults with developmental disorders or intellectual disability. There are limited studies which demonstrate CHD as a predictor of worse neurodevelopmental functioning among infants and toddlers with DS, and there is very little investigation of the impact CHD has on these abilities in school-aged children or older [25, 26]. Longitudinal studies comparing these outcomes in the two CHD groups (with and without DS) into adulthood do not exist. While there is limited direct evidence on employment rates specific to adults with CHD and a history of stroke or seizures, the association between neurodevelopmental impairment and reduced employment is better established in the broader stroke population [27]. There is a paucity of information on community engagement outcomes among adults with CHD specifically with a history of these neurologic conditions and outcomes among adults with both CHD and DS specifically are unknown [12]. It is unknown whether reported QOL differences exist in adults with both CHD and DS compared to either condition alone. Lastly, there is some evidence describing higher reported caregiver burden among primary caregivers to children with DS, with CHD being a significant associated factor; however, reports are nonspecific to CHD lesion type, severity, and have not included adults [28].

Individuals with both DS and CHD (DS + CHD) theoretically represent a higher-risk subgroup for worse functional outcomes in adulthood compared to those with DS alone. Community engagement, defined as paid employment and/or non-remunerative volunteerism, is a societal benchmark shown to be essential to QOL and well-being in adults, particularly those with disabilities [29, 30]. The World Health Organization defines QOL as “an individual’s perception of their position in life in the context of the culture and value systems in which they live and about their goals, expectations, standards and concerns.”^31^ We hypothesized that, compared to adults with Down syndrome alone (DS-CHD), those with congenital heart disease (DS + CHD) will exhibit lower community engagement, greater self- and proxy-reported neuropsychiatric conditions (specifically history of seizure, stroke, dementia, and/or mental health disorders), and poorer QOL.

## Methods

This was a cross-sectional survey assessment of adults with Down syndrome ages 18–45 years of age and their caregivers. Participants were recruited from three sources. A study advertisement was provided to regional Down syndrome organizations around South Carolina through community engagement and partnership in directly contacting these groups. Participants were additionally identified when seen for routine medical care by congenital cardiology centers, using the study advertisement provided to Pediatric Heart Network centers. Lastly, the study team collaborated with the DS-Connect: Down syndrome registry for subject recruitment. The DS-Connect was created by the NIH/NICHD Down Syndrome Consortium as a national health resource for people with Down syndrome and their families, researchers, and health care providers. It is a centralized, secure, web-based health registry to store and share health information and provide members access to new research studies [32]. At the time of study onset, 900 adults in DS-Connect had requested to be contacted if eligible to participate in a research study. This study was approved by the DS-Connect Research Review Committee to utilize the registry, and DS-Connect Registry Coordinators posted the study advertisement on their organization website and additionally directly contacted eligible members.

Participant eligibility criteria included a diagnosis of Down syndrome, being between 18 and 45 years of age, English-speaking, and having a participating caregiver to answer survey questions with the participant. Caregiver could include parent, other family member, unrelated guardian, or other (with specification requested). The study advertisement linked interested participants to a web-based form (REDCap) describing the research purpose, risks, benefits, and eligibility. Eligibility was confirmed by self-report in this web-based consent form. Given the minimal risk associated with anonymous survey responses, the limitations in assessing participant capacity and/or identifying Legally Appointed Representative designations in this manner, and the confidentiality of de-identified response storage, agreement to participate was requested from either the participant with DS or their caregiver. Once providing consent, participants were emailed the research study questionnaire and assigned a study number. Survey responses were de-identified to protect participant confidentiality, and email addresses were stored separately from survey responses within the study REDCap database. Following questionnaire completion, participants received a $25 e-gift card.

The study survey was a compilation of existing validated instruments and/or survey instruments which had been successfully utilized in this cohort. Additional question phrasing was modeled closely from validated survey formats. There were 6 components of the study questionnaire: demographic information, employment and volunteer history, WHOQOL-BREF-ID & Disabilities Module measuring quality of life for individuals with intellectual disability [31], Glasgow Depression Scale [33], health history (response types, including multiple choice, binary yes or no, and check-box), Zarit Burden Interview (short-form) assessing caregiver burden [34], and the employment and volunteer history. Questions included in the latter were modeled closely from Dr. Libby Kumin’s “Employment/Jobs Survey for People with Down Syndrome” questionnaire with her permission; this was a web-based survey developed in in conjunction with focus-group meetings and review by adults with Down syndrome and their parents [35]. Of note, question verbiage was modified when the COVID-19 pandemic occurred mid-study to specifically query subjects’ employment and volunteer activity pre-pandemic. The questionnaire was built and deployed via REDCap, a HIPAA-compliant secure web application for building and managing online survey and results, with a structured series of separate, linked survey sections and ability to save and complete in more than one setting by subjects and their caretakers to avoid survey fatigue. There were three survey response modes: self-report by the subject with DS independently, proxy-report with caregiver responses entirely, or collaborative report where the subject with DS and their caregiver complete portions of the survey together. Electronic completion of the study questionnaire was selected as the standard format given ease and accessibility, evidence that many individuals with DS possess strong computer skills and capabilities, and known difficulties with expressive language skills as would be required by telephone interview [35, 36]. The survey could also be mailed by subject request. Complete copy of the study questionnaire is available in Online Appendix 1, and the compilation of survey instruments and outcome measures are listed in Table 1.

**Table 1:** Study questionnaire components and outcome measures

Survey instrument	Outcome measure
Subject information: Demographics Health history Employment and Volunteer history	Basic characteristics Cardiac history NYHA classification by symptoms Stroke and/or seizure history Psychological comorbidities Employment and volunteer experience Education history Job-training activities
WHOQOL-BREF-ID & Disabilities Module (individual and proxy report options)	Quality of life
Glasgow Depression Scale	Mental health problems
Zarit Burden Interview	Caregiver burden

Of note, one study aim was to recruit a pilot subgroup among regional Down syndrome participants to complete both the study survey above and an additional in-person psychological assessment measuring cognitive ability, adaptive behavior skills, and executive function with an on-site study psychologist. This aim was complicated by the COVID-19 pandemic limiting safe and feasible in-person assessments during the study period, and not all portions of these instruments could be performed via telemedicine. Eventually, recruitment for this subgroup assessment was discontinued, and cognitive limitations were not assessed among subjects. A survey question was added to the study instrument to assess interest or willingness to come for neuropsychological assessments in future if possible.

To test the study hypothesis, group differences between DS + CHD and DS-CHD were investigated. The primary outcome was current employment rate. Additional outcomes included volunteerism, community engagement, neuropsychiatric conditions (history of seizure, stroke, mental health disorders), QOL, and caregiver burden. Statistical analyses were conducted using chi-square, Fisher’s exact, 2-sample *t* tests, and Wilcoxon rank sum tests, as appropriate. Analyses were performed with SAS v9.4 (SAS Institute, Cary, NC).

## Results

**Fig. 1:**
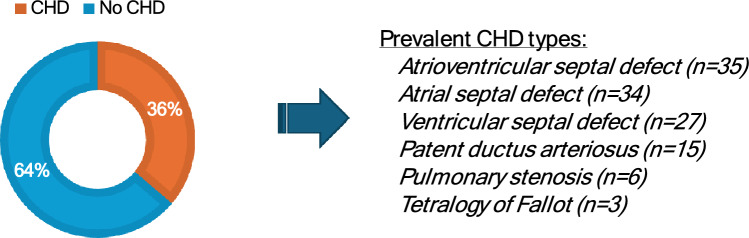
Proportion of participants with and without congenital heart disease (CHD), and specific congenital heart disease diagnoses

A total of 287 adults with Down syndrome and/or caregivers completed the study. The primary respondent was reportedly the person with Down syndrome (n = 120, 42%). Both caregiver and person with Down syndrome collaborated in 94 study respondents (33%), and the caregiver alone completed the entire study with proxy reports in 72 respondents (25%). The majority of caregivers endorsed being the parent of the adult with DS. Among respondents, 36% (n = 104) reported having CHD. Most CHD types were atrioventricular canal or a septal defect. Most of this group (n = 68, 65%) reported a history of prior cardiac surgery, predominantly within the first year of life (56%, n = 58). The mean ± SD total prior cardiac catheterizations in this group was 1.1 ± 1.3, and 33% (n = 34) reported taking a prescribed cardiac medication at time of study participation. The reported NYHA classification of the DS + CHD group was Class I or II for most respondents (n = 97, 93%), and a few subjects reported baseline cyanosis (7.7%, n = 8). Participant age, sex, Trisomy 21 type, and living arrangement responses were similar among the group of subjects with CHD and the group without; a comparison of demographics of each group and the reported CHD types is displayed in Table 2 and Fig. 1**.** Most participants lived with their parent(s), followed by living with siblings or other family, and less frequently in a group home, supported apartment, long-term assisted care facility, independently, or with spouse/partner.

**Table 2:** Demographics and specifics of Down syndrome diagnosis between the two groups

	DS + CHD (n = 104)	DS-CHD (n = 183)
Age (years)	27.0 ± 7.6	27.3 ± 8.2
Male sex	51.9% (n = 54)	58.5% (n = 107)
Complete Trisomy 21	87.5% (n = 91)	85.8% (n = 157)
Mosaic Trisomy 21	5.8% (n = 6)	6% (n = 11)
Marital status	Single (71.2%) Married (22.1%)	Single (78.1%) Married (15.3%)
Live with parents	76.9% (n = 80)	61.2% (n = 112)

Note: data not totaling to 100% reflects missing survey responses, “unknown” responses

**Fig. 2:**
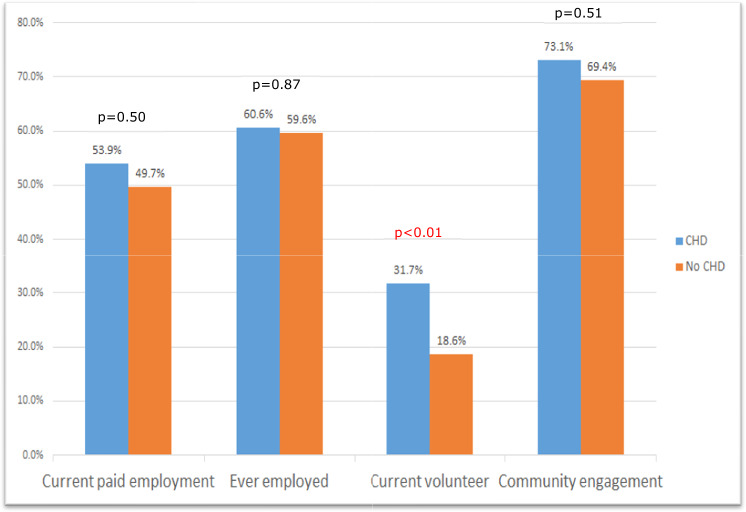
Differences in employment, volunteer participation, and community engagement between adults with both Down syndrome and CHD and those with DS alone

Among the entire DS cohort, the reported “current employment” rate pre-pandemic was 51.2% (n = 147), with 59.9% (n = 172) reported ever being employed. The total cohort “current volunteer participation” pre-pandemic was 23% (n = 67). Reported resources that helped prepare participants for paid or volunteer work include family, high school resources (counselor, transition program, or life skills course), job coach, vocational training programs, online learning or training resources, on-the-job training, church, and community workshops. There were no significant differences in rates of “current employment” (p = 0.50) or “ever-employment” (p = 0.87) between the groups of adults with and without CHD (Fig. 2**)**. Current pre-pandemic volunteer participation was reported in significantly more DS subjects with CHD than those without: 31.7% versus 18.6% (p = 0.01). “Community engagement” rates, comprising employment and/or volunteerism, were similar between the two groups: 73% in DS subjects with CHD and 69% of those without CHD (p = 0.51).

Psychiatric and mental health disorders were self-reported among 69% of the entire cohort (n = 198), from both Glasgow Depression Scale scores and “health history” intake responses. Specifically, this included self-reported autism spectrum disorder, depression, anxiety, obsessive–compulsive disorder, dementia, and/or a Glasgow Depression Scale score ≥ 13. The most prevalent issue reported was anxiety, followed by depression.

The WHO-QOL-BREF domain scores for all DS subjects were as follows: 70.3 ± 13.8 in Domain 1, 73.2 ± 13.3 in Domain 2, 65.0 ± 22.0 in Domain 3, and 86.1 ± 15.3 in Domain 4. The four domains assess QOL regarding physical health, psychological health, social relationships, and environment (respectively). Instrument domain mean scores range from 1 to 100, with higher scores representing higher QO. The WHO-QOL-DIS ID mean QOL score for all DS subjects by individual report (n = 221) was 66.6 ± 12.1 (instrument score range 1–100, with higher scores representing higher QOL). The proxy report (n = 66) mean QOL score for all DS subjects was 54 ± 13.0.

In a Chi-squared analysis, there were no significant differences in overall Glasgow Depression Scale scores between adults with DS + CHD and adults with DS alone (58.7% compared to 61.2%, respectively; p = 0.67) **(**Table 3**).** Similarly, *t* tests indicated that there was no significant difference in subject- or proxy-reported WHO-QOL-DIS ID mean QOL scores between the group with CHD compared to those without (subject [n = 221]: p = 0.52; proxy-reported [n = 66]: p = 0.25). There was no significant difference between Glasgow Depression scores or WHO-QOL-DIS-ID scores among adults with DS + CHD when examining cardiac surgery history as an independent variable (Glasgow Depression score: p = 0.39, WHO-QOL-DIS-ID: p = 0.23). Comparison of WHO-QOL-BREF Domain reports between the two groups was performed using the Wilcoxon Two-Sample Test, as the data were non-normally distributed (Table 2). There were no significant differences in WHO-QOL-BREF domain scores between adults with DS + CHD and adults with DS alone other than in Domain 4 (environment), with higher scores reported among those with CHD (89 ± 12.9 in DS + CHD compared to 84.4 ± 16.3 in DS alone). The Wilcoxon rank sum statistic for Domain 4 was 16,402, corresponding to a *p* value of 0.03.

**Table 3:** Differences in reported neurologic disorders, depression, quality of life, and caregiver burden between adults with CHD + DS and those with DS alone

	DS + CHD (n = 104)	DS-CHD (n = 183)	p
Stroke	6.7% (n = 7)	1.1% (n = 2)	< 0.01
Seizure	20.2% (n = 21)	7.1% (n = 13)	< 0.01
Depression (+ GDS score)	58.7% (n = 61)	61.2% (n = 112)	0.67
WHO-QOL-BREF Domain 1	70.4 ± 13.9	70.2 ± 13.8	0.82
WHO-QOL-BREF Domain 2	73.2 ± 12.7	73.2 ± 13.7	0.76
WHO-QOL-BREF Domain 3	65.6 ± 22.4	64.6 ± 21.9	0.80
WHO-QOL-BREF Domain 4	89.0 ± 12.9	84.4 ± 16.3	0.03
WHO-QOL-Dis *Subject report (n* = *221)*	65.7 ± 13.1	66.9 ± 11.6	0.52
WHO-QOL-Dis *Proxy score (n* = *66)*	51.8 ± 12.2	55.6 ± 14.3	0.25
Caregiver burden (ZBI mean)	12.3 ± 7.3	10.2 ± 9.0	0.03

WHO-QOL scores and ZBI scores presented as means ± standard deviation. WHO-QOL-BREF Domain scores were compared using the Wilcoxon Two-Sample Test

The entire cohort reported a history of prior stroke in 3.1% (n = 9) of respondents and seizure history in 11.9% (n = 34). Epilepsy was reported among 5.9% of all respondents (n = 17), and 1% reported a history of both seizure and stroke (n = 3). A Fisher’s exact test showed significantly higher rates of prior stroke in the group with CHD (6.7% compared to 1.1% without CHD, p < 0.01) and significantly higher rates of prior seizures in the group with CHD (20.2% compared to 7.1%, p < 0.01). When independently examined, histories of stroke or seizure in the entire cohort were not associated with increased depression scores, mean WHO-QOL-DIS scores, or rates of community engagement (p > 0.05 for each analysis).

The mean cohort caregiver burden score among all respondents was 11.0 ± 8.4 by Zarit Caregiver Burden Interview responses (scores ranging 0–48 with higher scores indicating greater caregiver burden). A *t* test demonstrated slightly higher caregiver burden scores among the group of CHD + DS adults (12.3 ± 7.3) compared to those without CHD (10.2 ± 9.0), p = 0.03.

A total of 116 subjects (40% of the entire cohort) recalled previously undergoing a formal intelligence quotient “IQ” test at some point. A significantly greater proportion of the group with CHD + DS (n = 68, 65%) reported prior “IQ” testing compared to subjects with DS alone (n = 48, 26%), *p* < 0.01. Overall, 50% (n = 143) of the entire cohort reported interest or willingness to come for neuropsychological testing in future, should this be an option. Willingness was significantly greater in the cohort of subjects with CHD + DS (74%) compared to those without CHD (38%) (*p* < 0.01 by Chi-square test), and this group also reported greater rates of prior formal psychological testing (65% compared to 26%, *p* < 0.01).

## Discussion

This is the first known study to engage adults with DS and their caregivers to assess productive functional status, neuropsychiatric conditions, and QOL, with specific isolation of comorbid CHD and its impact on these meaningful outcomes. This is a topic of increasing relevance given improving lifespan among individuals born with DS, with a parallel trend in pediatric cardiology with more adults than children living with CHD in the U.S. today [37]. Health and functional outcomes in this aging group with both DS and CHD has been highlighted more broadly in a recent Scientific Statement from the American Heart Association, underscoring the priority to better understand which comorbidities could merit greater resources for outcomes optimization [10]. Results from our cross-sectional survey assessment directly comparing two cohorts—DS only and CHD + DS—did not show that having CHD significantly impacts employment rates among adults with DS, nor did it have an association with worse QOL or depression (regardless of prior cardiac surgeries and greater rates of neurologic comorbidities). This novel information may convey optimism to families of individuals both with both DS and CHD, and it could serve to uncover existing interventions and systems that may be preventing worse outcomes to then apply more broadly.

Employment and other components of community participation are demonstrated to be vital to QOL and psychological well-being in adulthood, including individuals with disabilities and their families [29]. However, individuals with disabilities have significantly lower employment rates than those without (24.2% compared to 65.8% according to the most recent Bureau of Labor Statistics) [38]. When more specifically examining employment outcomes among adults with DS, as Kumin et al. initially surveyed in 2015 with input in questionnaire design directly from adults with DS and their families, this group reported a more favorable “current paid employment rate” of 57% in comparison to broader groups with disabilities [35]. The similar employment rate of 54% among adults with both DS and CHD in this study cohort has not previously been reported and is largely encouraging, suggesting that cardiac history seems to not add detriment.

Volunteer work also should not be overlooked as a potentially meaningful way that individuals with DS contribute to their communities. Adults with DS have reported significantly higher rates of volunteerism than the population of typical adults (42% in the Kumin et al. cohort, 23% in our overall study group). The higher volunteer participation rates among study respondents with CHD plus DS compared to DS alone could reflect more motivated caregivers to identify opportunities or earlier involvement in volunteer organizations in childhood and adolescence. However, an alternate argument is that this may reflect the difficulty in finding enough hours of paid employment or adequate daytime care for these adults. This topic could be further explored in future studies. Regardless, these reported rates of community participation not only have meaning to patients and their families but also for health care providers who engage with these individuals often throughout the entirety of their childhood and can influence future transition planning and goals. This data could impart similar meaning to educators, policymakers, rehabilitation counselors, and employers to broaden the trajectory for community engagement opportunities for adults with DS beyond education attainment.

Children born with CHD are at increased risk for neurocognitive and psychologic problems at all stages of early development, with increased vulnerability demonstrated among those with genetic abnormalities [39–41]. Cognitive challenges may include difficulties in memory, attention, executive functions (planning, problem solving, flexibility), processing speed, language, and visuospatial skills. Many tertiary children’s heart centers have incorporated neurodevelopmental assessments and therapies among infants and children with complex CHD seemingly at highest risk [42]. Additional threats to neurocognitive abilities in children with CHD (with or without genetic syndromes) include higher rates of neurologic conditions, including perioperative stroke and seizures, which are risks that interestingly persist throughout the lifespan. Seizures occur with increased frequency in adult CHD patients, particularly those with more complex CHD, and stroke incidence in young adults is nearly eleven times that of the general population [22, 23, 43, 44]. In general, both stroke and seizure have been associated with lower executive functioning, employment challenges, and worse QOL [12, 45, 46] With mutually well-described patterns of neurocognitive pathology and learning impairments among individuals with DS, regular developmental assessments throughout childhood and educational assistance is recommended. Individuals with DS also exhibit more frequent rates of stroke and seizures, the former less well understood with most reports in case series; presumably having certain types of CHD or prior cardiac surgery may heighten one’s risk for either issue [47]. This investigation is the first to confirm this prediction among adults with both DS and CHD. The more intriguing finding in this work is that these higher rates of stroke and seizure were not associated with reduced community engagement or lower QOL compared to adults with without these prior neurologic comorbidities. Interestingly, there was a significant difference in the reported QOL domain of “environment” with lower perceived QOL among adults with both CHD + DS compared to those without CHD. This raises additional questions regarding differences in the surroundings, resources and access to services or activities, and functional abilities translating to a satisfactory daily environment in the CHD + DS group. Some adults with CHD have physical activity restrictions, or there is a fear among caregivers that activity should be restricted, and perhaps this contributes. Additional study is needed to better understand relevant factors.

Increased caregiver burden scores among respondents of subjects with CHD + DS compared to DS alone may reflect both the medical complexity of CHD and the additional support needs for community engagement. However, it should be noted that both groups reported only “mild” degree of burden on the instrument score, which ranges 1–48 and each group’s mean score of < 13. The study questionnaire did not further characterize the caregiver’s actual role in the subject’s daily life, and there may be substantial variability in responsibility among caregiver respondents.

Adults with DS reported a high prevalence of mental health problems, regardless of CHD status. This is consistent with existing evidence describing conditions specifically associated with DS, including Alzheimer’s disease, attention deficit disorder, autism, anxiety, and depression [3, 48–50]. Higher risks for cognitive and neurodevelopmental deficiencies exist among adults with CHD as well, postulated for years and recently formally evaluated and demonstrated among those with more complex CHD types [13]. This study originally aimed to perform a similar pilot assessment of cognitive ability and adaptive behaviors in adults with DS compared to those with complex CHD to evaluate CHD-mediated differences in this special young adult cohort. However, sample size was unable to be achieved largely related to COVID-19 pandemic-related testing space and safety concerns impacting enrollment. The aim may be more feasible in a post-pandemic period given that half of the entire cohort (50.2%, n = 143) self-reported willingness to complete neuropsychological testing.

Limitations to the study include the validity of self-reported data and mixed response groups, including exclusive self-report, exclusive proxy-report by caregiver, and collaborative report. A variety of questionnaire response options were permitted to account for the wide range of functional ability among adult subjects with DS; however, mixing reporting modes can introduce measurement inconsistency, response bias, and comparability problems. Additional study limitations included hacking of web-based questionnaires resulting in subsequent restriction of study advertisement, consent in vulnerable populations, and selection bias in more motivated subjects and families with greater willingness to participate. There may be outcomes differences among the age ranges of adult subjects with DS included (18–45 years), and future studies could stratify into more specific categories. Study enrollment began immediately prior to the COVID-19 pandemic, and while survey instrument questions were re-phrased to query subjects’ community engagement status “pre-pandemic,” this still may have impacted subject recruitment and/or responses given lower rates of employment and volunteer participation nationally at this time. Similarly, the COVID-19 pandemic was associated with an increased prevalence of mental health problems and potentially greater caregiver burden with closures of adult day programs and other occupational facilities and resources, and this may have confounded survey responses.

## Conclusion

This is the first study engaging adults with DS and their caregivers to evaluate the impact CHD may have on productive community functions, neuropsychiatric conditions, and QOL, as well as reported caregiver burden. Interestingly, CHD was not associated with reduced employment or community engagement, despite significantly greater neurologic comorbidities. Similar QOL and mental health issues were reported in adults with DS regardless of having CHD. A generally low level of caregiver burden was reported in all respondents. These findings may convey optimism to families of children born with both health issues and could be used by providers and policymakers to better understand helpful or protective systems for this group.

## Supplementary Information

Below is the link to the electronic supplementary material.

Supplementary file1 (PDF 130 KB)

## Data Availability

No datasets were generated or analysed during the current study.

## Abbreviations

DS: Down syndrome
CHD: Congenital heart disease
QOL: Quality of life
