# Supplementary material for: Reported Impacts of Congenital Heart Disease on Functional Outcomes in Adults with Down Syndrome
Source: Pediatr Cardiol. 2025 Jul 31;47(5):1933–41. doi: 10.1007/s00246-025-03979-2 (PMC13144255; doi:10.1007/s00246-025-03979-2)
Supplement: Supplementary file 1 — Supplementary file1 (PDF 130 KB) [file 246_2025_3979_MOESM1_ESM.pdf]

The Effect of Congenital Heart Disease on Employment, Neuropsychological Outcomes, and Quality of Life in Adults with Down syndrome: Introduction

Thank you for enrolling in this study. As mentioned before, your answers are confidential and will not be directly linked to information which will identify you. Please answer all the survey questions to the best of your ability.

Make sure you begin the study surveys when you are seated with your parent or caregiver. Each survey section will state who should answer the questions. You may take breaks and return to the study surveys whenever you wish. There are 6 total survey sections.

Please save the original email containing the survey link in order to return to the survey; alternatively, if you click "Save and Return," your survey will be emailed to you to easily return to where you left off.

Once you have finished all parts of the survey, you will be prompted to enter one email address for the study team to send you a $25 Amazon e-gift card as a thank-you for your participation. This e-gift card will be emailed to the email address you provide within 1-3 days of finishing the study survey.

If you encounter problems or have questions, please feel free to contact our study team for assistance:
Shameeka Bowman: 843-792-8317 (bowm@musc.edu)
Dr. Stephanie Gaydos: gaydoss@musc.edu

The first several questions ask some basic demographic information about you, the participant with Down syndrome, and your parent or caregiver.

These can be answered by either the adult with Down syndrome, the parent/caregiver, or both.

---

1. What is the participant's Down syndrome diagnosis?

- ◯ Complete Trisomy 21
- ◯ Mosaic Trisomy 21
- ◯ Translocation Down syndrome
- ◯ Mosaic translocation Down syndrome
- ◯ Partial Trisomy 21 (only a part of chromosome 21 is present in 3 copies)
- ◯ Not tested
- ◯ Unsure

---

2. What is the age of the adult with Down syndrome?

____

3. Gender of the adult with Down syndrome

◯ Male
◯ Female

4. Relation of caregiver to the participant with Down syndrome

◯ Parent
◯ Sibling
◯ Spouse
◯ Other family member
◯ Unrelated guardian
◯ Other

4a. Specify:

____

5. Age of caregiver:

____

6. Highest level of education of primary caregiver

◯ Less than 9th grade
◯ 9th to 12th grade, no diploma
◯ High school graduate, GED, or alternative
◯ Some college, no degree
◯ Associate degree
◯ Bachelor's degree (e.g., BA, AB, BS, BBA)
◯ Post-bachelor's degree (e.g., MA, MS, MBA, MD, DDS, JD, PhD)

7. Marital status of the participant with Down syndrome

◯ Single
◯ Married
◯ Living with partner
◯ Separated
◯ Divorced
◯ Widowed

8. Living circumstances and support of the adult with Down syndrome (select the one that best describes your situation)

◯ Lives with parents (may include siblings)
◯ Lives with sibling
◯ Lives with other family member
◯ Lives in a group home
◯ Lives in a supported apartment
◯ Lives with spouse or with partner
◯ Lives independently
◯ Lives in a long-term care facility
◯ Other

8a. Specify:

____

9. Are you currently in ill or in poor health?

◯ Yes
◯ No

10. If you had the option to come to a nearby clinic for psychologist testing of things like IQ, memory, specific areas of brain function and behavior, would you be interested or willing to do this?
(Optional, for potential future studies)

◯ Yes
◯ No

11	11. For our record-keeping, please select "Yes" to indicate that you are completing this survey at a time when COVID-19 is prevalent in the U.S. (which we realize may impact some survey answers).	◯ Yes

"Quality of Life" Survey

This survey section should be answered directly by the adult with Down syndrome, if possible. The questions ask how you feel about your quality of life, health, or other areas of your life. It is just about you-- you and your life. There are 39 questions.

If you would like some help to click your answer choice, it is OK to ask someone to do this for you.

Can you (and/or are you willing to) answer these survey questions yourself?

If so, click "Yes" to continue.

If you cannot answer the questions, click "No" to be directed to a "Proxy" survey where your parent or guardian will answer the questions on your behalf.

*(Note, if you begin answering questions and change your mind about the most appropriate respondent, you may return to this question to change your answer. You would then be directed to restart the survey with the new respondent choice).

- ◯ Yes
- ◯ No

QUALITY OF LIFE SURVEY:
These questions should be answered by the adult with Down syndrome.

This assessment asks how you feel about your quality of life, health or other areas of your life. It is just about you and your life. Please keep in mind what is important to you; what makes you happy; your hopes and dreams, and your worries or concerns.

Please answer all the questions. If you are unsure about which answer to give to a question - if it is hard to pick an answer - please choose the one that seems nearest or most appropriate. This can often be the first thing that comes into your mind. Some questions include an example to help you think about your answer.

There are no right or wrong answers - just answer what is true for you. Please think about your life in the last two weeks.

For example, thinking about the last two weeks, a question might ask:

"Do you get the kind of support that you need from others?"
For example, do you get the kind of help you need from other people?

In this item, the question has an example. You should choose the answer that best fits your opinion about the kind of support (or help) you got from others over the last two weeks. So you would select "Moderately" if the support (or help) you got met your needs moderately. Alternatively, you would choose "Not at all" if the support you got over the last two weeks did not meet your needs at all.

Please read each question, think about your feelings, and choose the answer for each question that gives the best answer for you.

You may find it helpful to look at these 'smiley faces' that add a visual guide (a picture) to the answer options.

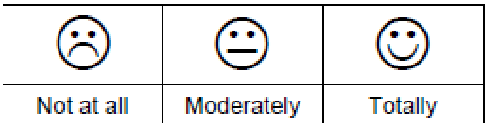

Please think about your life in the last two weeks:
The first two questions ask about your life and health overall.

1 Are you satisfied with your life?

◯ Not at all ◯ A little ◯ Moderately ◯ Mostly ◯ Totally

2 Are you satisfied with your health?

◯ Not at all ◯ A little ◯ Moderately ◯ Mostly ◯ Totally

The following questions ask about how you have felt about certain things, how much you have been able to do certain things, or how satisfied you have been about various aspects of your life over the last two weeks.

3 Does (physical) pain stop you from doing what you need to do?

◯ Not at all ◯ Moderately ◯ Totally

4 Do you need any medical treatment to help you in your daily life?
For example, medicines.

◯ Not at all ◯ Moderately ◯ Totally

5 Do you enjoy your life?

◯ Not at all ◯ Moderately ◯ Totally

6 Do you feel your life has meaning?
For example, do you feel your life is important and has a purpose?

◯ Not at all ◯ Moderately ◯ Totally

7 Are you able to think clearly?
For example, are you able to pay attention, and think carefully about things?

◯ Not at all ◯ Moderately ◯ Totally

8 Do you feel safe in your daily life?
For example, at home and in the places you go to during the day.

◯ Not at all ◯ Moderately ◯ Totally

9 Do you feel the local area you live in is healthy?
For example, thinking about the noise, the traffic, the pollution, the weather.

◯ Not at all ◯ Moderately ◯ Totally

10 Do you have enough energy for everyday life?
For example, are you able to do things through the day without feeling tired?

◯ Not at all ◯ Moderately ◯ Totally

11 Are you able to accept the way your body looks?

◯ Not at all ◯ Moderately ◯ Totally

12 Do you have enough money for the things you need?

◯ Not at all ◯ Moderately ◯ Totally

13 Are you able to get the information that you need in your day-to-day life?
For example, is it easy to find out about things you need to know in your daily life?

◯ Not at all ◯ Moderately ◯ Totally

14 Do you get the chance to do leisure activities?
For example, do you get the chance to do things you enjoy in your spare time?

◯ Not at all ◯ Moderately ◯ Totally

15 Are you able to get around OK in the house and outside?

◯ Not at all ◯ Moderately ◯ Totally

16 Are you satisfied with your sleep?

◯ Not at all ◯ Moderately ◯ Totally

17 Are you satisfied with your ability to do your daily activities?
For example, looking after yourself, washing, dressing, eating.

◯ Not at all ◯ Moderately ◯ Totally

18 Are you satisfied with your ability to work?
For example, to do your job, or your daily activities.

◯ Not at all ◯ Moderately ◯ Totally

19 Are you satisfied with yourself as a person?
For example, with the kind of person you are, in what you do, how you spend your time, your friendships, your achievements.

◯ Not at all ◯ Moderately ◯ Totally

20 Are you satisfied with your personal relationships?
For example, how do you get along with the people in your life, your friends, your family, the people you live with.

◯ Not at all ◯ Moderately ◯ Totally

21 Are you satisfied with your sex life, or your relationship with your partner?
For example, your husband/wife, boyfriend/girlfriend.

◯ Not at all ◯ Moderately ◯ Totally

22 Are you satisfied with the support you get from your friends?

◯ Not at all ◯ Moderately ◯ Totally

23 Are you satisfied with what your home is like?
For example, thinking about your home and the place you live in.

◯ Not at all ◯ Moderately ◯ Totally

24 Are you satisfied with your access to health services?
For example, is it easy to see the doctors, nurses, or other staff who look after you when you are unwell?

◯ Not at all ◯ Moderately ◯ Totally

25 Are you satisfied with the transport you can use?
For example, how you get to the places you go to (e.g. by bus, car, taxi etc.)?

◯ Not at all ◯ Moderately ◯ Totally

26 Do you feel very unhappy, sad, worried or depressed?

◯ Not at all ◯ Moderately ◯ Totally

The next question asks about your disability overall.

27 Does your disability have a negative (bad) effect on your day-to-day life?

◯ Not at all ◯ A Little ◯ Moderately ◯ Mostly ◯ Totally

The following questions ask about how you have felt about certain things, how much certain things have applied to you, and how satisfied you have been about various parts of your life over the last two weeks.

28 Do you feel that some people treat you unfairly?

◯ Not at all ◯ Moderately ◯ Totally

29 Do you need someone to stand up for you when you have problems?

◯ Not at all ◯ Moderately ◯ Totally

30 Do you worry about what might happen to you in the future?
For example, thinking about not being able to look after yourself, or being a burden to others in the future.

◯ Not at all ◯ Moderately ◯ Totally

31 Do you feel in control of your life?
For example, do you feel in charge of your life?

◯ Not at all ◯ Moderately ◯ Totally

32 Do you make your own choices about your day-to-day life?
For example, where to go, what to do, what to eat.

◯ Not at all ◯ Moderately ◯ Totally

33 Do you get to make the big decisions in your life?
For example, like deciding where to live, or who to live with, how to spend your money.

◯ Not at all ◯ Moderately ◯ Totally

34 Are you satisfied with your ability to communicate with other people?
For example, how you say things or get your point across, the way you understand others, by words or signs.

◯ Not at all ◯ Moderately ◯ Totally

35 Do you feel that other people accept you?

◯ Not at all ◯ Moderately ◯ Totally

36 Do you feel that other people respect you?
For example, do you feel that others value you as a person, and listen to what you have to say?

◯ Not at all ◯ Moderately ◯ Totally

37 Are you satisfied with your chances to be involved in social activities?
For example, meeting friends, going out for a meal, going to a party etc.

◯ Not at all ◯ Moderately ◯ Totally

38 Are you satisfied with your chances to be involved in local activities?
For example, being part of what is happening in your local area or neighborhood.

◯ Not at all ◯ Moderately ◯ Totally

39 Do you feel that your dreams, hopes and wishes will happen?
For example, do you feel you will get the chance to do the things you want, or get the things you wish for, in your life?

◯ Not at all ◯ Moderately ◯ Totally

Thank you. You have finished this portion of the study survey. Please click "Submit" to move onto the next section of questions.

Remember, if you need to take a break and return to the survey at a later time, you may do so at any point by clicking "Save and Return," then using the link emailed to you.

QUALITY OF LIFE SURVEY: Proxy Version
These questions should be answered by the parent, guardian, or legally authorized representative of the person with Down syndrome.

You are being asked to complete this assessment on behalf of someone else (a proxy assessment). This may be because that person cannot answer the questions (e.g. as a result of illness or disability), or because the views of another individual who knows the person well are being sought also.

In order to complete a proxy assessment, you must know well the person for whom you are responding. For example, you might be the person's partner, a member of their family, a close friend, a health or social care worker, a health professional or their advocate (a person formally or legally appointed to help them). You should answer the questions as you think the person for whom you are responding would answer, using all your knowledge and experience of that person and their life.

This assessment asks how the person for whom you are responding feels about their quality of life, health or other areas of their life. It is about them, and their life, rather than about you. Please keep in mind what is important to the person for whom you are responding; what makes them happy; their hopes and dreams, and their worries or concerns.

Please answer all the questions. If you are unsure about which answer to give to a question- if it is hard to pick an answer, please choose the one that seems nearest or most appropriate. This can often be the first thing that comes into your mind. Some questions include an example to help you think about your answer.

There are no right or wrong answers - just answer what you believe is true. Please think about the life of the person in the last two weeks.

For example, thinking about the last two weeks, a question might ask:

"Does he/she get the kind of support that he/she needs from others?"
For example, does he/she get the kind of help he/she needs from other people?

In this item, the question has an example. You should select the answer that best fits what the person for whom you are responding would say about the kind of support (or help) they got from others over the last two weeks. So you would select "Moderately" if the support (or help) the person got met their needs moderately. Alternatively, you would select "Not at all" if the person for whom you are responding would say that the support they got over the last two weeks did not meet his/her needs at all.

Please read each question, think about the feelings of the person for whom you are responding, and select the answer for each question that gives the best answer for him/her.

You may find it helpful to look at the 'smiley faces' that add a visual guide (a picture) to the answers.

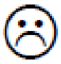	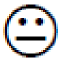	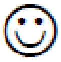
Not at all	Moderately	Totally

Please think about the life of the person for whom you are responding in the last two weeks.

The first two questions ask about his/her life and health overall.

---

1 Is he/she satisfied with his/her life?

○ Not at all ○ A Little ○ Moderately ○ Mostly ○ Totally

---

2 Is he/she satisfied with his/her health?

○ Not at all ○ A Little ○ Moderately ○ Mostly ○ Totally

The following questions ask about how he/she has felt about certain things, how much he/she has been able to do certain things, or how satisfied he/she has been about various aspects of his/her life over the last two weeks.

---

3 Does (physical) pain stop him/her from doing what he/she needs to do?

○ Not at all ○ A Little ○ Moderately ○ Mostly ○ Totally

---

4 Does he/she need any medical treatment to help him/her in his/her daily life?
For example, medicines.

○ Not at all ○ A Little ○ Moderately ○ Mostly ○ Totally

---

5 Does he/she enjoy his/her life?

○ Not at all ○ A Little ○ Moderately ○ Mostly ○ Totally

---

6 Does he/she feel his/her life has meaning?
For example, does he/she feel his/her life is important and has a purpose?

○ Not at all ○ A Little ○ Moderately ○ Mostly ○ Totally

---

7 Is he/she able to think clearly?
For example, is he/she able to pay attention, and think carefully about things?

○ Not at all ○ A Little ○ Moderately ○ Mostly ○ Totally

8 Does he/she feel safe in his/her daily life?
For example, at home and in the places he/she goes to during the day.

◯ Not at all ◯ A Little ◯ Moderately ◯ Mostly ◯ Totally

9 Does he/she feel the local area he/she lives in is healthy?
For example, thinking about the noise, the traffic, the pollution, the weather.

◯ Not at all ◯ A Little ◯ Moderately ◯ Mostly ◯ Totally

10 Does he/she have enough energy for everyday life?
For example, is he/she able to do things through the day without feeling tired?

◯ Not at all ◯ A Little ◯ Moderately ◯ Mostly ◯ Totally

11 Is he/she able to accept the way his/her body looks?

◯ Not at all ◯ A Little ◯ Moderately ◯ Mostly ◯ Totally

12 Does he/she have enough money for the things he/she needs?

◯ Not at all ◯ A Little ◯ Moderately ◯ Mostly ◯ Totally

13 Is he/she able to get the information that he/she needs in his/her day-to-day life?
For example, is it easy to find out about things he/she needs to know in his/her daily life?

◯ Not at all ◯ A Little ◯ Moderately ◯ Mostly ◯ Totally

14 Does he/she get the chance to do leisure activities?
For example, does he/she get the chance to do things he/she enjoys in his/her spare time?

◯ Not at all ◯ A Little ◯ Moderately ◯ Mostly ◯ Totally

15 Is he/she able to get around OK in the house and outside?

◯ Not at all ◯ A Little ◯ Moderately ◯ Mostly ◯ Totally

16 Is he/she satisfied with his/her sleep?

◯ Not at all ◯ A Little ◯ Moderately ◯ Mostly ◯ Totally

17 Is he/she satisfied with his/her ability to do his/her daily activities?
For example, looking after himself/herself, washing, dressing, eating.

◯ Not at all ◯ A Little ◯ Moderately ◯ Mostly ◯ Totally

18 Is he/she satisfied with his/her ability to work?
For example, to do his/her job, or his/her daily activities?

◯ Not at all ◯ A Little ◯ Moderately ◯ Mostly ◯ Totally

19 Is he/she satisfied with himself/herself as a person?
For example, with the kind of person he/she is, in what he/she does, how he/she spends his/her time, his/her friendships, his/her achievements.

◯ Not at all ◯ A Little ◯ Moderately ◯ Mostly ◯ Totally

20 Is he/she satisfied with his/her personal relationships?
For example, how he/she gets along with the people in his/her life, his/her friends, his/her family, the people he/she lives with.

◯ Not at all ◯ A Little ◯ Moderately ◯ Mostly ◯ Totally

21 Is he/she satisfied with his/her sex life, or his/her relationship with his/her partner?
For example, his/her husband/wife, boyfriend/girlfriend.

◯ Not at all ◯ A Little ◯ Moderately ◯ Mostly ◯ Totally

22 Is he/she satisfied with the support he/she gets from his/her friends?

◯ Not at all ◯ A Little ◯ Moderately ◯ Mostly ◯ Totally

23 Is he/she satisfied with what his/her home is like?
For example, thinking about his/her home and the place he/she lives in?

◯ Not at all ◯ A Little ◯ Moderately ◯ Mostly ◯ Totally

24 Is he/she satisfied with his/her access to health services?
For example, is it easy to see the doctors, nurses or other staff who look after him/her when he/she is unwell?

◯ Not at all ◯ A Little ◯ Moderately ◯ Mostly ◯ Totally

25 Is he/she satisfied with the transport he/she can use?
For example, how she gets to the places he/she goes to (e.g. by bus, car, taxi etc).

◯ Not at all ◯ A Little ◯ Moderately ◯ Mostly ◯ Totally

26 Does he/she feel very unhappy, sad, worried or depressed?

◯ Not at all ◯ A Little ◯ Moderately ◯ Mostly ◯ Totally

The next question asks about his/her disability overall.

27 Does he/she feel that his/her disability has a negative (bad) effect on his/her day-to-day life?

◯ Not at all ◯ A Little ◯ Moderately ◯ Mostly ◯ Totally

The following questions ask about how he/she has felt about certain things, how much certain things have applied to him/her, and how satisfied he/she has been about various parts of his/her life over the last two weeks.

28 Does he/she feel that some people treat him/her unfairly?

◯ Not at all ◯ A Little ◯ Moderately ◯ Mostly ◯ Totally

29 Does he/she feel that he/she needs someone to stand up for him/her when he/she has problems?

◯ Not at all ◯ A Little ◯ Moderately ◯ Mostly ◯ Totally

30 Does he/she worry about what might happen to him/her in the future?
For example, thinking about not being able to look after him/herself, or being a burden to others in the future.

◯ Not at all ◯ A Little ◯ Moderately ◯ Mostly ◯ Totally

31 Does he/she feel in control of his/her life?
For example, does he/she feel in charge of his/her life?

◯ Not at all ◯ A Little ◯ Moderately ◯ Mostly ◯ Totally

32 Does he/she make his/her own choices about his/her day-to-day life?
For example, where to go, what to do, what to eat.

◯ Not at all ◯ A Little ◯ Moderately ◯ Mostly ◯ Totally

33 Does he/she get to make the big decisions in his/her life?
For example, like deciding where to live, or who to live with, how to spend his/her money.

◯ Not at all ◯ A Little ◯ Moderately ◯ Mostly ◯ Totally

34 Is he/she satisfied with his/her ability to communicate with other people?
For example, how he/she says things or gets his/her point across, the way he/she understands others, by words or signs.

◯ Not at all ◯ A Little ◯ Moderately ◯ Mostly ◯ Totally

35 Does he/she feel that other people accept him/her?

◯ Not at all ◯ A Little ◯ Moderately ◯ Mostly ◯ Totally

36 Does he/she feel that other people respect him/her?
For example, does he/she feel that others value him/her as a person and listen to what he/she has to say?

◯ Not at all ◯ A Little ◯ Moderately ◯ Mostly ◯ Totally

37 Is he/she satisfied with his/her chances to be involved in social activities?
For example, meeting friends, going out for a meal, going to a party, etc.

◯ Not at all ◯ A Little ◯ Moderately ◯ Mostly ◯ Totally

38 Is he/she satisfied with his/her chances to be involved in local activities?
For example, being part of what is happening in his/her local area or neighborhood.

◯ Not at all ◯ A Little ◯ Moderately ◯ Mostly ◯ Totally

39 Does he/she feel that his/her dreams, hopes and wishes will happen?
For example, does he/she feel he/she will get the chance to do the things he/she wants, or get the things he/she wishes for, in his/her life?

◯ Not at all ◯ A Little ◯ Moderately ◯ Mostly ◯ Totally

39 Thank you. You have finished this portion of the study survey. Please click submit to move onto the next section of questions.

Remember, if you need to take a break and return to the survey at a later time, you may do so at any point using the link emailed to you.

Glasgow Depression Scale

Like the last section, this survey section questions should be answered directly by the participant with Down syndrome if possible. The questions ask about your mood and your mental health. There are 20 questions.

**NOTE: These survey questions are not diagnostic tools for depression or other mental health problems. If you are concerned by question content/answers, please call your primary care doctor for further discussion.

If you would like some help to click your answer choice, it is OK to ask someone to do this for you.

Can you (and/or are you willing to) answer these survey questions yourself?

-If so, click "Yes" to continue.
-If you CANNOT answer the questions, click "No" to be directed to a "Proxy" survey where your parent or guardian will answer the questions on your behalf.

◯ Yes
◯ No

All questions refer to "in the last 1 week." Additional questions are listed as examples to better explain the question's meaning.

---

1 Have you felt sad?
(Have you felt upset? Have you felt miserable? Have you felt depressed?)

◯ Never/ No
◯ Sometimes
◯ Always/ A lot

---

2 Have you felt as if you are in a bad mood?
(Have you felt bad-tempered? Have you felt as if you want to shout at people?)

◯ Never/ No
◯ Sometimes
◯ Always/ A lot

---

3 Have you enjoyed the things you have done?
(Have you had fun? Have you enjoyed yourself?)

◯ Never/ No
◯ Sometimes
◯ Always/ A lot

---

4 Have you enjoyed talking to people and being with other people?
(Have you liked having people around you? Have you enjoyed other people's company?)

◯ Never/ No
◯ Sometimes
◯ Always/ A lot

---

5 Have you made sure you have washed yourself, worn clean clothes, brushed your teeth and combed your hair?
(Have you taken care of the way you look? have you looked after your appearance?)

◯ Never/ No
◯ Sometimes
◯ Always/ A lot

6 Have you felt tired during the day?
(Have you gone to sleep during the day? Have you found it hard to stay awake during the day?)

◯ Never/ No
◯ Sometimes
◯ Always/ A lot

7 Have you cried?

◯ Never/ No
◯ Sometimes
◯ Always/ A lot

8 Have you felt you are a horrible person?
(Have you felt others don't like you?)

◯ Never/ No
◯ Sometimes
◯ Always/ A lot

9 Have you been able to pay attention to things (such as watching TV)?
(Have you been able to concentrate on things, like television programs? Are you able to watch your favorite program from start to finish?)

◯ Never/ No
◯ Sometimes
◯ Always/ A lot

10 Have you found it hard to make decisions?
(Have you found it hard to decide what to wear, or what you would like to eat, or do? Have you found it hard to choose between two things?)

◯ Never/ No
◯ Sometimes
◯ Always/ A lot

11 Have you found it hard to sit still?
(Have you fidgeted when you are sitting down? Have you been moving about a lot, like you can't help it?)

◯ Never/ No
◯ Sometimes
◯ Always/ A lot

12 Have you been eating too much, or too little?
(Do people say you should eat more/less?)

◯ Never/ No
◯ Sometimes
◯ Always/ A lot

13 Have you found it hard to get a good night's sleep?
(Have you found it hard to fall asleep at night? Have you woken up in the middle of the night and found it hard to get back to sleep? Have you woken up too early in the morning?)

◯ Never/ No
◯ Sometimes
◯ Always/ A lot

14 Have you felt that life is not worth living?
(Have you wished you could die? Have you felt you do not want to go on living?)

◯ Never/ No
◯ Sometimes
◯ Always/ A lot

15 Have you felt as if everything is your fault?
(Have you felt as if people blame you for things? Have you felt that things happen because of you?)

◯ Never/ No
◯ Sometimes
◯ Always/ A lot

16 Have you felt that other people are looking at you, talking about you, or laughing at you?
(Have you worried about what other people think of you?)

◯ Never/ No
◯ Sometimes
◯ Always/ A lot

17 Have you become very upset if someone says you have done something wrong or you have made a mistake?
(Do you feel sad or feel like crying if someone tells you that you made a mistake?)

◯ Never/ No
◯ Sometimes
◯ Always/ A lot

18 Have you felt worried?
(Have you felt nervous? Have you felt tense/wound up/on edge?)

◯ Never/ No
◯ Sometimes
◯ Always/ A lot

19 Have you thought that bad things keep happening to you?
(Have you felt that nothing nice ever happens to you anymore?)

◯ Never/ No
◯ Sometimes
◯ Always/ A lot

20 Have you felt happy when something good happened?
(If nothing good happened in the past week, then would you feel happy if someone gave you a nice present?)

◯ Never/ No
◯ Sometimes
◯ Always/ A lot

Thank you. You have finished the survey section. Click "Submit" to continue to the next set of questions, or save to return to the survey at a later time.

If these survey questions and answers have made you concerned about your mood and mental health, please contact your primary care doctor. If you are in need of immediate assistance, please dial 911 or the National Suicide Prevention Lifeline at 1-(800) 273-8255.

The following questions ask how you think your adult son or daughter with Down syndrome (or the adult with Down syndrome under your guardianship or representation) has been in the last week. This person will be referred to as 'X' in the following questions. There is no right or wrong answer.

Please select the answer you feel best describes X in the last week.

1 Has X appeared depressed?

◯ Never/ no
◯ Sometimes/ a little
◯ Always/ a lot

2 Has X been more physically or verbally aggressive than usual?

◯ Never/ no
◯ Sometimes/ a little
◯ Always/ a lot

3 Has X avoided company or social contact?

◯ Never/ no
◯ Sometimes/ a little
◯ Always/ a lot

4 Has X looked after his/her appearance?

◯ Never/ no
◯ Sometimes/ a little
◯ Always/ a lot

5 Has X spoken or communicated as much as he/she used to?

◯ Never/ no
◯ Sometimes/ a little
◯ Always/ a lot

6 Has X cried?

◯ Never/ no
◯ Sometimes/ a little
◯ Always/ a lot

7 Has X complained of headaches or other aches and pains?

◯ Never/ no
◯ Sometimes/ a little
◯ Always/ a lot

8 Has X still taken part in activities which used to interest him/her?

◯ Never/ no
◯ Sometimes/ a little
◯ Always/ a lot

9 Has X appeared restless or fidgety?

◯ Never/ no
◯ Sometimes/ a little
◯ Always/ a lot

10 Has X appeared lethargic or sluggish?

◯ Never/ no
◯ Sometimes/ a little
◯ Always/ a lot

11 Has X eaten too little/ too much?

◯ Never/ no
◯ Sometimes/ a little
◯ Always/ a lot

12 Has X found it hard to get a good night's sleep?

For example, has X had difficulty falling asleep when going to bed at night, waking in the middle of the night and finding it hard to get back to sleep again, and/or been waking very early in the morning and finding it hard to get back to sleep?

◯ Never/ no
◯ Sometimes/ a little
◯ Always/ a lot

13 Has X been sleeping during the day?

◯ Never/ no
◯ Sometimes/ a little
◯ Always/ a lot

14 Has X said that he/she does not want to go on living?

◯ Never/ no
◯ Sometimes/ a little
◯ Always/ a lot

15 Has X asked you for reassurance?

◯ Never/ no
◯ Sometimes/ a little
◯ Always/ a lot

16 Have you noticed any change in X recently?

◯ Never/ no
◯ Sometimes/ a little
◯ Always/ a lot

16a Please explain what changes you have noticed, in either mood or behavior.

Thank you. You have finished the survey section. Click "Submit" to continue to the next set of questions, or save to return to the survey at a later time.

If these survey questions and answers have made you concerned about X's mood and mental health, please contact his or her primary care doctor. If you are in need of immediate assistance, please dial 911 or the National Suicide Prevention Lifeline at 1-(800) 273-8255.

Health History

This survey section can be completed by the adult participant with Down syndrome and/or the participant's parent or caregiver. Questions are about the participant's medical history, with invited assistance from the participant's parent or caregiver.

Proxy completion by the participant's parent or guardian should be indicated in the first question below.

Please answer the questions to the best of your knowledge.

*If you are completing this questionnaire for the participant with Down syndrome, please answer all questions with information about the participant only. "You" refers to the participant with Down syndrome.

Who is filling out this survey?

◯ Adult participant with Down syndrome
◯ Parent or family member
◯ Unrelated caregiver/ legal guardian
◯ Both

Have you ever been diagnosed with a heart problem?

◯ Yes
◯ No

What is the name of the heart problem? (Select all that apply)

☐ Aortic valve stenosis
☐ Atrial septal defect (ASD)
☐ Atrioventricular septal defect (AVSD) or Atrioventricular canal (AV canal)
☐ Bicuspid aortic valve
☐ Coarctation of aorta
☐ Hypoplastic left heart syndrome (HLHS)
☐ Pulmonary atresia
☐ Pulmonary valve stenosis
☐ Tetralogy of Fallot (TOF)
☐ Transposition of the great arteries (TGA)
☐ Ventricular septal defect (VSD)
☐ Truncus arteriosus
☐ Single ventricle (double inlet left ventricle)
☐ Patent ductus arteriosus (PDA)
☐ Pulmonary hypertension
☐ Arrhythmia (heart rhythm problem)
☐ Other
☐ Do not know / not sure

Please provide name or description:

____

Next, we will ask you questions about any surgeries you may have had on your heart. Heart surgery will result in scars on the middle of your chest, side, or back. Surgeries that occur after the first surgery may use the same scar or create a new scar.

Have you ever had any cardiac or heart surgery? (Select all that apply)

(*Note, this is different from a heart catheterization).

- ☐ None
- ☐ Yes, surgery for a congenital heart defect in the first year of life
- ☐ Yes, surgery for a congenital heart defect after the first year of life
- ☐ Cardiac surgery for other reason later in life
- ☐ Unsure

How many total cardiac or heart surgeries have you had?

____

Have you ever had a heart catheterization ("cath") before? (Select all that apply).

- ☐ None
- ☐ Yes, a cath for a congenital heart defect in the first year of life
- ☐ Yes, a cath for a congenital heart defect after the first year of life
- ☐ Heart catheterization for other reason later in life
- ☐ Unsure

How many total heart catheterizations ("caths") have you had?

____

Next, we will ask you about symptoms which may be related to your heart.

Select one of the choices below that best describes your ability to do physical activity:

- ◯ I can perform all physical activity without getting short of breath or tired, or having palpitations.
- ◯ I get short of breath or tired, or have palpitations when performing more strenuous activities. For example, walking on steep inclines or walking up several flights of steps.
- ◯ I get short of breath or tired, or have palpitations when performing day to day activities. For example, walking on the flat ground.
- ◯ I feel breathless at rest, and am mostly housebound. I am unable to carry out any physical activity without getting short of breath or tired, or having palpitations.

Is your baseline oxygen level lower than normal due to your heart condition? (for example, have you been told that you have "cyanosis" or "hypoxia"?)

- ◯ Yes
- ◯ No

Are you currently prescribed medicine(s) for your heart?

- ◯ Yes
- ◯ No

Next we will ask you about other health problems you may have been diagnosed with. Please answer to the best of your ability.

What other health problems do you currently have, or had in the past? Select all that apply.

☐ Celiac disease
☐ Thyroid problem
☐ Leukemia
☐ Other cancer
☐ Diabetes
☐ Obesity
☐ Problems with spine
☐ Sleep apnea
☐ Hearing loss
☐ Vision loss, cataracts, or other eye problems
☐ Autism spectrum disorder
☐ Depression
☐ Anxiety
☐ Obsessive-compulsive disorder
☐ Alzheimer's dementia (or other dementia)
☐ Stroke
☐ Regression or loss of previously attained skills (speech-language, social, self-help or motor skills)
☐ Multiple dental caries (cavities)
☐ Wears dentures
☐ Other

Specify which other health problems you have:

____

Have you ever had a formal IQ (intelligence) test?

◯ Yes
◯ No

Please select the age of the most recent IQ test you have had.

◯ Between birth to age 5 years
◯ 6-10 years of age
◯ 11-15 years of age
◯ 16-20 years of age
◯ 21-25 years of age
◯ Older than 25 years of age

Please select the score of your most recent IQ test.

◯ IQ greater than 115
◯ IQ 100 - 115
◯ IQ 85 - 100
◯ IQ 70-84
◯ IQ 50-69
◯ IQ 35-49
◯ IQ 20-34
◯ IQ Below 20
◯ I don't know

Next, we will ask you questions about history of stroke or seizure (or both).

Have you had any of the following brain studies? (Select all that apply, to the best of your knowledge)

☐ Brain MRI scan (magnetic resonance imaging)
☐ Brain CT scan (computed tomography)
☐ Cranial ultrasound
☐ EEG recording (electroencephalography)
☐ Not sure
☐ None of the above

If you had a brain imaging study, what was the reason for obtaining the study? (Select all that apply, to the best of your knowledge)

☐ Newborn brain ultrasound for bleeding or enlarged ventricles
☐ New onset of seizures
☐ New onset of regression
☐ Symptoms of dementia
☐ Head injury or loss of consciousness
☐ Other neurological symptoms
☐ For scientific research
☐ Unsure
☐ Brain imaging has not been performed

Have you ever been told you have (or had) a stroke?

○ Yes
○ No

When did you have a stroke? Please select all that apply

☐ Less than 1 year of age
☐ Age 1-5 years old
☐ Age 6-17 years old
☐ Age 18 years or older

What type of stroke did you have?

○ Ischemic
○ Hemorrhagic (brain bleeding)
○ Both
○ Don't know/ not sure

Please select any problems that you had after your stroke(s). Select all that apply.

☐ More difficulty speaking
☐ Changes in mood
☐ Motor function problems (for example, weakness or clumsiness in arms or legs, difficulty walking, droopy side of face, trouble swallowing)
☐ Changes in personality/behavior
☐ No noticeable problems
☐ Don't know/ not sure

Have you ever had a seizure?

○ Yes
○ No

Have you been told you had a seizure disorder (epilepsy)?

◯ Yes
◯ No

If so, around what age were you diagnosed with a seizure disorder (epilepsy)?

◯ Less than 1 year old
◯ Age 1-5 years old
◯ Age 6-17 years old
◯ Age 18 years or older

How long has it been since your last seizure?

◯ Less than 1 month
◯ Between 1-12 months
◯ Between 1-5 years
◯ More than 5 years

Are you currently prescribed medicine(s) for seizures?

◯ Yes
◯ No

Employment

The purpose of this survey is to collect information on employment, unemployment, volunteer jobs, and education. The survey should be completed by parents/guardians and their adult children with Down syndrome. Please answer to the best of your ability.

Please answer about your job/volunteer situation BEFORE COVID-19, with the knowledge that many people’s circumstances have changed since this event.

If you are completing this questionnaire for the subject, please answer all questions with information about the subject with Down syndrome only. "You" refers to the subject.

Who is filling out this survey section?

◯ Participant with Down syndrome
◯ Caregiver
◯ Both

What is the highest degree or grade you have completed?
(Note: "You" refers to the person with Down syndrome in each of these questions)

◯ Never attended school or only attended kindergarten
◯ 8th grade or less
◯ More than 8th grade, but did not graduate from high school
◯ High school graduate
◯ GED (General Education Development Test for high school equivalent)
◯ Certificate of attendance from high school
◯ Went to a business, trade, or vocational school after high school
◯ Went to college, but did not graduate
◯ Associate degree
◯ Bachelor's degree
◯ Professional training beyond a four-year college or university
◯ Don't know/not sure

In elementary, junior high, or high school were you ever in a special education program? Please select all that apply:

☐ Special education
☐ Advanced placement
☐ Homebound education
☐ My school did not have these types of programs available
☐ Other:
☐ None

Please describe what other type of program you participated in:

BEFORE COVID-19, were you:

◯ Working at a paid job
◯ Working at a volunteer job
◯ Working at a combination of paid and volunteer jobs
◯ Self-employed or have your own business
◯ Not working at all

How long have you been unemployed?

◯ Less than 6 months
◯ Between 6 months to 1 year
◯ More than 1 year

Have you ever been employed?

◯ Yes
◯ No

How many years ago were you employed?

____

For how long were you employed at your most recent job?

◯ Less than 6 months
◯ Between 6 months to 1 year
◯ More than 1 year

Reason for unemployment: (check all that apply)

☐ Looking for a job
☐ Laid off
☐ Fired
☐ In a training program
☐ Choosing to volunteer instead
☐ Not interested in working
☐ Cannot work because of health problems
☐ Still in high school
☐ Attending a post-secondary program
☐ Inadequate transportation
☐ Other

Please specify what other reason you have for being unemployed:

____

BEFORE COVID-19, total number of hours you were working each week at your paid job? (On average)

____

Total number of hours you are working each week at your volunteer job? (On average).

____

Work type (most recent job):

☐ Competitive employment
☐ Sheltered work setting
☐ Have your own business/self-employed
☐ Other

Where did you work? (most recent job)

☐ Fast food restaurant
☐ Catering
☐ Supermarket
☐ Big box store (such as Target/Walmart)
☐ Landscaping/outdoors
☐ Janitorial/cleaning
☐ Office skills job
☐ Other

Please describe:

BEFORE COVID-19, how many different paid jobs did you have?

____

BEFORE COVID-19, what was your pay per hour? (optional)

____

How did you find this job (most recent job)?

☐ Rehabilitation agency
☐ Employment agency
☐ Newspaper ads
☐ Internship
☐ Parent, friends and family contacts
☐ Religious group
☐ Other

Please specify the other way you found this job:

BEFORE COVID-19, how long had you been working at this job?

◯ Under 6 months
◯ 6-11 months
◯ 1-2 years
◯ 3-4 years
◯ 4-5 years
◯ More than 5 years

Did you have training in high school that prepared you for this job?

◯ Yes
◯ No

Did you have training after high school that prepared you for this job?

☐ Job skills program
☐ Post-secondary program
☐ On the job training
☐ Other
☐ No I did not

Please explain what other training:

BEFORE COVID-19, were you doing volunteer work?

◯ Yes
◯ No

How long had you worked at this volunteer job?

◯ Under 6 months
◯ 6-11 months
◯ 1-2 years
◯ 3-4 years
◯ 4-5 years
◯ More than 5 years

Did you have training in high school that prepared you for this volunteer work?

◯ Yes
◯ No

Did you have training after high school that prepared you for this volunteer work?

☐ Job skills program
☐ Post-secondary program
☐ On the job training
☐ Other
☐ No I did not have any training

Please explain what other training:

How did you find the volunteer work?

☐ School counselor
☐ Rehabilitation agency counselor
☐ Employment agency
☐ Parent, family and friends contacts
☐ Religious group
☐ Other

Please specify:

In high school, did you participate in a job-training program?

◯ Yes
◯ No

If so, how helpful was this?

◯ Not very helpful
◯ Somewhat helpful
◯ Very helpful

Have you ever had a job coach?

◯ Yes
◯ No

If so, how helpful was this?

◯ Not very helpful
◯ Somewhat helpful
◯ Very helpful

What has been the most helpful resource to prepare you for your paid or volunteer work?

BEFORE COVID-19, how did you feel about your paid job(s)?

◯ Happy/ Satisfied
◯ No strong feelings
◯ Unhappy/ Dissatisfied

Caregiver Burden

This is the last section of questions within the study survey. These questions should be answered by the participant's parent/caregiver only. If you are not currently with your parent/caregiver, you can close the survey here and return to this link when your caregiver is available.

Questions ask about your perceived "caregiver burden," including the stress and implications that can be involved with caring for another person.

Indicate how often you experience the feelings listed in each question by selecting the answer that best corresponds to the frequency of these feelings.

---

1. Do you feel that because of the time you spend with your relative (or person under your care) that you don't have enough time for yourself?

◯ Never
◯ Rarely
◯ Sometimes
◯ Quite Frequently
◯ Nearly Always

---

2. Do you feel stressed between caring for your relative and trying to meet other responsibilities (work/family)?

◯ Never
◯ Rarely
◯ Sometimes
◯ Quite Frequently
◯ Nearly Always

---

3. Do you feel angry when you are around the relative?

◯ Never
◯ Rarely
◯ Sometimes
◯ Quite Frequently
◯ Nearly Always

---

4. Do you feel that your relative currently affects your relationship with family members or friends in a negative way?

◯ Never
◯ Rarely
◯ Sometimes
◯ Quite Frequently
◯ Nearly Always

---

5. Do you feel strained when you are around your relative?

◯ Never
◯ Rarely
◯ Sometimes
◯ Quite Frequently
◯ Nearly Always

6. Do you feel that your health has suffered because of your involvement with your relative?

◯ Never
◯ Rarely
◯ Sometimes
◯ Quite Frequently
◯ Nearly Always

7. Do you feel that you don't have as much privacy as you would like because of your relative?

◯ Never
◯ Rarely
◯ Sometimes
◯ Quite Frequently
◯ Nearly Always

8. Do you feel that your social life has suffered because you are caring for your relative?

◯ Never
◯ Rarely
◯ Sometimes
◯ Quite Frequently
◯ Nearly Always

9. Do you feel that you have lost control of your life since your relative's illness?

◯ Never
◯ Rarely
◯ Sometimes
◯ Quite Frequently
◯ Nearly Always

10. Do you feel uncertain about what to do about your relative?

◯ Never
◯ Rarely
◯ Sometimes
◯ Quite Frequently
◯ Nearly Always

11. Do you feel you should be doing more for your relative?

◯ Never
◯ Rarely
◯ Sometimes
◯ Quite Frequently
◯ Nearly Always

12. Do you feel you could do a better job in caring for your relative?

◯ Never
◯ Rarely
◯ Sometimes
◯ Quite Frequently
◯ Nearly Always

Thank you. You have finished all portions of the study survey! Please click "Submit" below.

The research team will send you a $25 Amazon e-gift card as a 'Thank you' for participating within 1-3 days of submitting this survey. This will be sent to the email address you initially provided to receive this study survey.

If you do not receive the email or have any questions or problems with your e-gift card, please contact our study team for assistance:
bowm@musc.edu
gaydoss@musc.edu

After submitting this form, you will have the opportunity to print a copy of this page for your records and/or future use to contact our study team.

Scoring

Total Module Score ______________________________
